# Cyber-Threat Detection System Using a Hybrid Approach of Transfer Learning and Multi-Model Image Representation

**DOI:** 10.3390/s22155883

**Published:** 2022-08-06

**Authors:** Farhan Ullah, Shamsher Ullah, Muhammad Rashid Naeem, Leonardo Mostarda, Seungmin Rho, Xiaochun Cheng

**Affiliations:** 1 School of Software, Northwestern Polytechnical University, 127 West Youyi Road, Beilin District, Xi’an 710072, China; 2 School of Electronic Information and Artificial Intelligence, Leshan Normal University, Leshan 614000, China; 3 Computer Science Department, Camerino University, 62032 Camerino, Italy; 4 Department of Industrial Security, Chung-Ang University, Seoul 06974, Korea; 5 Department of Computer Science, Middlesex University, London NW4 4BT, UK

**Keywords:** malware detection, malware visualization, transfer learning, network traffic, explainable AI, cyber security

## Abstract

Currently, Android apps are easily targeted by malicious network traffic because of their constant network access. These threats have the potential to steal vital information and disrupt the commerce, social system, and banking markets. In this paper, we present a malware detection system based on word2vec-based transfer learning and multi-model image representation. The proposed method combines the textual and texture features of network traffic to leverage the advantages of both types. Initially, the transfer learning method is used to extract trained vocab from network traffic. Then, the malware-to-image algorithm visualizes network bytes for visual analysis of data traffic. Next, the texture features are extracted from malware images using a combination of scale-invariant feature transforms (SIFTs) and oriented fast and rotated brief transforms (ORBs). Moreover, a convolutional neural network (CNN) is designed to extract deep features from a set of trained vocab and texture features. Finally, an ensemble model is designed to classify and detect malware based on the combination of textual and texture features. The proposed method is tested using two standard datasets, CIC-AAGM2017 and CICMalDroid 2020, which comprise a total of 10.2K malware and 3.2K benign samples. Furthermore, an explainable AI experiment is performed to interpret the proposed approach.

## 1. Introduction

We have entered the “mobile era” with the advent of sophisticated technologies and smartphones becoming increasingly common. Traditional cognitive platforms that power desktop computers are being displaced by smartphones and tablets with massive computational capability. Apps that were previously only available on high-end desktop computers are now available on a variety of mobile platforms. Mobile phones have evolved into devices that allow users to conduct online transactions, communicate with friends, and play games [1]. The number of apps accessible for download on the Google Play Store expanded between 2009 and 2017. The Google Play Store (https://www.statista.com/statistics/266210/number-of-available-applications-in-thegoogle-play-store (accessed on 20 February 2022)) had more than 3.5 million apps as of December 2017, an increase from slightly more than 1 million in July 2013. Furthermore, mobile network data are rapidly growing, and cloud services are hastening this process. Android has the largest market share in terms of mobile operating systems. The rapid expansion of Android has spawned a thriving developer community. Hundreds of millions of apps can be downloaded in seconds from various Android marketplaces. As smartphones and tablets become more popular, the number of mobile malware threats targeting them grows [2]. The number of ransomware attacks nearly doubled in 2021, as reported by the National Computing Centre (NCC) (https://www.nccgroup.com/uk/ (accessed on 3 July 2022)) Group. For instance, the number of reported ransomware attacks increased by 92.7% between 2020 and 2021, from 1389 to 2690. Network-based malware is becoming more sophisticated and difficult to combat. This means that we must now deal with everything from network-based malware to internet services that are protected by mobile devices. Furthermore, adversaries are becoming increasingly capable of creating malware that can avoid traditional sandboxing [3]. It is critical to establish a strong network-based malware classification and detection mechanism.

Mobile malware detection solutions can be classified as static, dynamic, or traffic-based [4]. Several previous studies used a static technique to detect vulnerabilities and malware in Android apps. The complexity and diversity of the required codes make this method challenging. Many dynamic approaches attempt to change the operating system of the phone to monitor and recover sensitive information. These strategies are effective, but they necessitate a significant amount of computing power to investigate all possible app patterns [5]. Several malware detection algorithms focus on network traffic generated by Android apps. Malware can be identified by abnormal network behavior patterns. This type of malware detection technology is very useful because the vast majority of Android malware performs harmful behaviors via network activity. To perform malicious acts, malware must communicate with a host system via the network. These traces allow different types of malwares to be tracked and identified. Furthermore, compared to previous methods, developing a network-based malware detection system is less difficult. For instance, such a method can be used at an entry point or gateway without overburdening the mobile device. These solutions are solely based on data generated by consumers, ensuring that users have access to desired mobile apps. Furthermore, other than granting rights to the identifying service, these solutions require no user engagement [6,7]. The goal of network traffic-based approaches is to discover distinctive features of malware that may be used to classify it accurately.

### 1.1. Problem Statement

**Figure 1:**
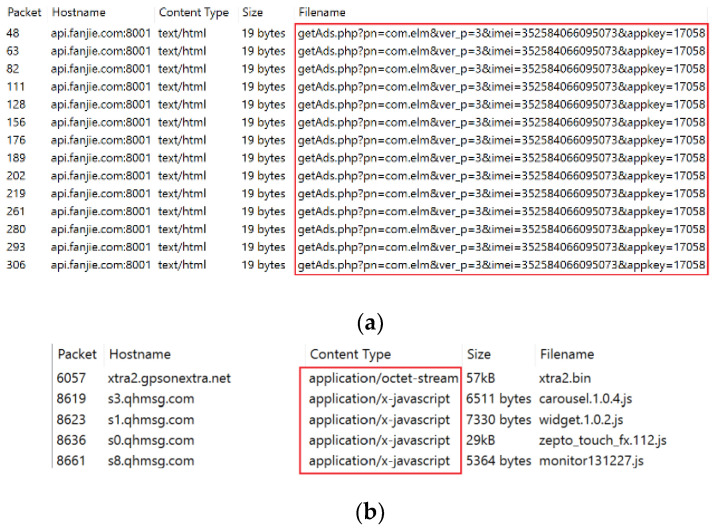
Malicious behaviors of adware and riskware network traffic. (**a**) Adware, (**b**) riskware.

Network traffic malware may employ several malicious URL scripts to affect a target Android app. Text-based feature analysis can identify potentially harmful scripts in terms of behavioral segmentation. Figure 1 depicts the malicious activities of adware and riskware. Riskware can embed malicious bytes required for remote code execution. For instance, “application/x-javascript” is incorporated in network traffic to be executed on a remote device to prevent normal access. Similarly, Adware is a type of malware that hides on the target system and displays advertisements. Some adware also monitors internet activity to serve relevant advertisements. Such behavior cannot be achieved solely through image visualization. However, text-based analysis is associated with several issues, such as code obfuscation, insertion, reordering, etc. Image-based malware classification is widely used because it can collect all types of structural information such as memory, process, header, etc. As a result, visual images can be used to retrieve any type of dynamic or obfuscated data. However, it can alter the overall structure of network traffic files, rendering it impossible to target a specific script, such as a malicious script, URL, etc. Furthermore, this method is completely reliant on image attributes. For instance, a hacker can attack a malware image, affecting overall classification performance. As a result, we combined text-based features to detect potential malicious scripts and textural image features to detect other dangerous behaviors, such as memory or resource utilization. A hybrid approach can efficiently use and classify malware and benign files.

### 1.2. Research Contributions

In this paper, we propose a novel method for analyzing and characterizing network-based malware. The HTTP and TCP flows are filtered from encrypted communications for broad analysis. Then, word2vec is utilized to capture the trained vocab features. Then, the network-based byte stream is converted to an image. The text-based and visual features are combined for effective malware classification. We observed that these two sorts of features complement one other and that combining them can increase the detection rate of malware. The main contributions of the paper are as follow:

- A malware classification and detection system is proposed using a hybrid approach of transfer learning and texture features. The proposed method adopts the benefits of both methods, i.e., textual and visual analysis.
- An explainable AI experiment is designed to interpret and validate the proposed approach.

The remainder of this paper is organized as follows. In Section 2, we describe the related work, and in Section 3, we describe the proposed method. In Section 4, we thoroughly discuss the experiments, and in Section 5 we present our conclusions.

## 2. Related Work

Several studies [8,9] had demonstrated how the Android platform protects infected target devices using a variety of security measures, including permission processes. However, individuals have to be adequately qualified with respect to security concerns to benefit from admin privilege protection. These limits imposed by excessive reliance on the customer enable Android malware to infiltrate and proliferate via portable devices. The majority of such analyzers examine aspects such as permissions and potentially unwanted programs to determine whether an application is suspicious or not. Antivirus apps protect computers against malware threats. However, malicious software is always evolving and expanding. As a consequence, malware detection methods need improvement. Several malware detection systems can currently decipher malicious activity in APK files without executing them.

Sanz et al. [10] developed a static approach that accurately classifies infections by capturing an app’s uses-permission and uses-feature details, as well as the user’s permission information for log files. The proposed method achieved 86.41% classification accuracy. Puerta et al. [11] used the same approach to detect malware using the Drebin dataset and achieved 96.05% accuracy. Liu et al. [12] proposed a two-phase malware detection method. The first phase involves analyzing the app’s Manifest.xml document, which provides requested permissions. The second phase is to preprocess the APK file using APK tools to obtain the smali code. The smali code may contain details about asserted permissions, including API calls, which may be used to detect malicious acts. The suggested technique has a detection performance of 98.6%. Shanshan et al. [13] proposed an HTTP- and TCP-based malware detection system for abnormal network assessment. The network device replicates the portable app’s data flow. All information retrieval and malware identification take place on the web, utilizing the fewest resources possible. Network-based characteristics and neural network models are coupled to identify mobile malware with an accuracy of 97.89%. Aresu et al. [14] investigated HTTP-based datagrams produced by Android apps when they interact with distant malicious servers. It also applies a grouping method of producing profiles from several malware variants. These markers are then employed to determine unusual operations. Wang et al. [15] developed the TextDroid methodology, which divides an HTTP content flow into special symbols and then generates n-gram sequences to study the layout of the resulting attributes. TextDroid also collects sequential information to feed into a learning algorithm for malware identification. This text-based technique achieved a classification score of 76.99%. Shanshan et al. [16] presented data traffic as a concept for detecting mobile malware. Natural language processing (NLP) tools are used to exploit an HTTP text file for knowledge representation. The next step is to detect malware by inspecting the linguistic characteristics of network data. The presented scheme has a classification performance of 95%. Data from TCP and HTTP traffic features are extracted by TrafficAV and compared to each other using a C4.5 decision tree for accuracy comparison. However, this method does not integrate TCP and HTTP network traces for the machine learning model. It provides a malware detection rate of 98.16% based on HTTP flows [17]. Johann et al. [18] proposed a WebEye framework that generates feasible HTTP traffic on its own, enriches captured traffic with detailed information, and classifies records as malicious or benign using various classifiers, with an accuracy rate of 89.52%.

Numerous studies [19,20] using deep learning to classify malware have produced promising results. A perceptron called the multi-layer perceptron (MLP) [21] works with other perceptrons stacked in multiple layers to categorize malware. A CNN [22] is primarily used to deal with texture features from malware images in order to classify malware. Gradient boosting [23] uses an ensemble of weak prediction models, usually decision trees, to classify malware. A temporal convolutional network (TCN) [24] is influenced by convolutional architectures, which combine easiness, vector autoregression prediction, and enormously long memory for malware classification. A general meta-approach to machine learning called ensemble learning combines the predictions from various models to improve malware classification performance [25]. Chen et al. [26] proposed a CNN model for categorizing mobile apps that relies on HTTP logs. The use of CNN speeds up the selection of features, resulting in more precise traffic detection outputs. The presented method achieved an identification rate of 98%. David et al. [27] introduced the DeepSign method, which is based on deep belief networks. It is capable of producing immutable, concise definitions of malware activities, which can enable it to effectively differentiate nearly all current malware variants with an accuracy of 98.6%. Shanshan et al. [28] introduced an HTTP-based malware classification method. A multi-view neural network is used to detect destructive behavior with varying levels of penetration. This method can be used to focus on certain attributes of input parameters by allocating continuous attention to features. The highest and lowest accuracy rates are 98.81% and 89.33%, respectively.

## 3. Proposed Method

**Figure 2:**
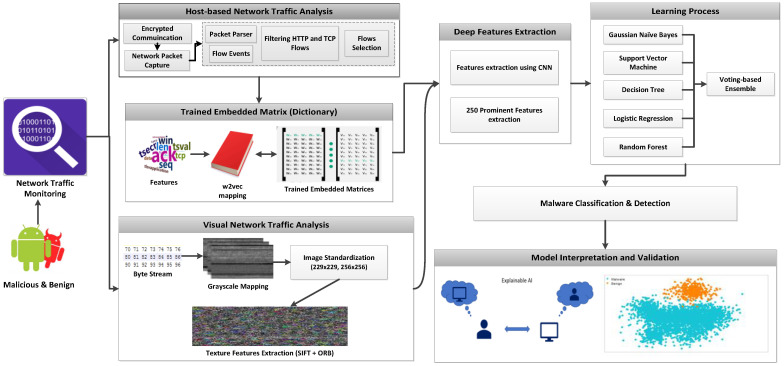
Cyber-threat detection system using a hybrid approach of word2vec-based transfer learning and visual representation.

Figure 2 explains the architectural framework of the proposed method. Android network traffic is monitored and extracts encrypted communication in the form of packet capture files. The network traffic in two ways, i.e., via textual or visual features.

### 3.1. Network Trace Collection

#### 3.1.1. Network Data Preprocessing

HTTP traffic is used because it is the most widely used protocol for global communication. HTTP headers contain data that can be used to detect malicious behavior. However, because mobile apps communicate via HTTP, critical information cannot be obtained. To address this issue, we analyze TCP streams with HTTP traces from packet capturing (PCAP) files. PCAP files are source documents generated by network communication. Such files contain network traffic information and are used to assess the underlying information exchange between malicious nodes. Furthermore, they make network traffic management and network activity detection easier. A packet parsing method that filters secure communication and extracts HTTP and TCP flows is developed. The packet parser algorithm is used to filter the PCAP file, as shown in Algorithm 1.
**Algorithm 1:** Packet Parser Algorithm**Input**: Packet Capturing Files (PCAP)**Output**: TCP, HTTP as output filesStep 1: Set $P=\{p_{1},\;p_{2},\;\ldots ,\;p_{n}\}$, where *P* is a packetStep 2: $Filter\;\left(P\right)=P^{\prime }$
Step 3: Compute PCAP from $P^{\prime }$, where $P^{\prime }=\left(\mathrm{IP},\mathrm{TCP},\mathrm{HTTP},\ldots ,n\right)$Step 4: Select NF from PCAP, where NF is the required network flowsStep 5: Display/select HTTP + TCP

HTTP traces include source IP, destination IP, port, host address, source info, bytes, packet length, frame length, and TTL. The source information section includes GET, POST, and URLs, such as “www.yahoo.com” (accessed on 5 December 2021). TCP flows provide three-way handshake information, including uploaded and downloaded bytes and total packet numbers during different sessions. Such information can be filtered to capture meaningful information, preserving the actual semantics. We developed a semantic tokenizer that can filter such information. The main steps taken during data preprocessing are as follow:

- Remove consecutively identical features from input sequences to avoid duplicated data.
- Short sequences may not include enough information to identify the relevant network traffic and are eliminated from the dataset.
- Because different sequence lengths confuse neural network models, unifying sequence length is critical for malware classification. This approach uses a preset sequence length (L) to balance the lengths. Sequences greater than L keep their first L names, but those shorter than L are unified through zero padding.

#### 3.1.2. Transfer Learning with Word2vec

**Figure 3:**
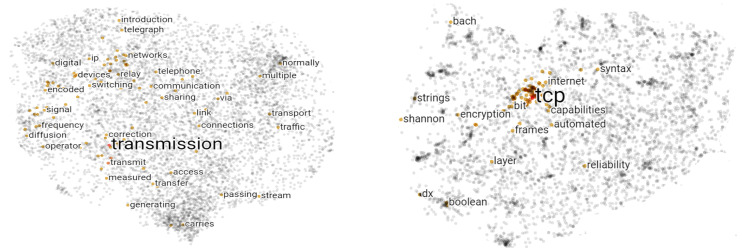
Visualization of trained features (transmission, tcp) using word2vec and TensorFlow.

The neural network operates through the use of vectors. Network traffic is represented by a fixed-size vector (L), and a one-hot vector can be employed. However, its scope is limited by the variety of features. This method is unsuitable for learning large datasets. Therefore, a reduced and meaningful vector is required. Word2vec [29] satisfies these criteria. Our goal is to construct a dense vector for each network element that records its contexts in a big dataset. Geometric techniques can be used on network vectors to detect their logical similarities, i.e., intruders use the same web address or TCP conversation for the same victim. Figure 3 demonstrates word2vec with TensorFlow embedding. In our situation, word2vec is used to mine trained vocab features from legitimate and malignant apps. The embedding word model output is a matrix, K × A, where K is the embedding vector size, and A is the number of unique network features. The encoded-word vector can be trained independently for malware classification [30]. The embed vectors are trained with 8-dimensionally for small datasets and with 1024-dimensionally for large datasets. We selected 300 dimensions for HTTP and TCP. Higher-dimensional embeddings require more data for finer word correlations. The trained vocab features are extracted from word2vec using dynamic fine tuning. Using this procedure, each feature is transferred to a large number of vectors with the same meaning. As a result, this mapping function allows for multiple interpretations of the same feature, which may change over time. Algorithm 2 shows trained feature extraction process from network flows.
**Algorithm 2:** Trained Feature Mining**Input**: HTTP and TCF flows**Output**: Trained featuresStep 1: Select HTTP and TCP flowsStep 2: Tokenize and filter HTTP and TCP flows = clean featuresStep 3: Apply fine-tune embedding
     • Dynamic word2vec = train featureStep 4: Extraction = train featureStep 5: Compute trained files = mining trained filesStep 6: Finish

### 3.2. Texture Feature Collection

**Figure 4:**
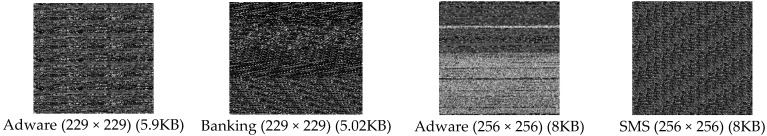
A chunk of malware images (229 × 229, 256 × 256) extracted from network traffic.

Considering that malware is frequently changed to circumvent static and dynamic identification, we analyzed a malware detection system based on texture properties. This technique detects the malware as a whole by turning the malware into an image and obtaining the textural features. It is not necessary to collect malware fingerprints or use reverse engineering tools. This strategy is effective against antidetection technologies, such as signature modification and dynamic feature detection evasion. We developed a malware-to-image conversion algorithm capable of retrieving images from PCAP files. The eight-bit vectors are retrieved from network traffic first and then processed to produce grayscale malware images. The image sizes are then standardized to 229 × 229 and 256 × 256. Figure 4 depicts a collection of malware images for adware (229 × 229), banking (229 × 229), adware (256 × 256), and SMS (256 × 256). A large PCAP size is transformed to a smaller image size. For instance, the PCAP is converted from megabytes to kilobytes in the image. As a result, it may be possible to reduce computation power. The extraction of texture features is illustrated in Algorithm 3. The extracted network bytes from PCAP files are utilized to mine texture features. These network bytes are represented as images. The texture features are then extracted from these images by combining SIFT and ORB descriptors. SIFT identifies key points or local features within a texture. These steady characteristics can be used for image comparison, object tracking, and scene recognition, among other applications. SIFT consistently outperforms ORB, although ORB is the fastest method. When the angle of rotation is 90 degrees, ORB and SIFT exhibit similar behavior [31]. In order to take advantage of both techniques, we combined SIFT and ORB descriptors to obtain pixel values representing texture features.
**Algorithm 3:** Texture Feature Mining**Input**: Network traffic (Bytes)**Output**: Texture featuresStep 1: Compute $B=\left\{B_{1},\;B_{2},\;\ldots .,\;B_{n}\;\right\}$, where B is for BytesStep 2: Compute I, where I is imageStep 3: Decompose I in $SS_{1}\;\&\;SS_{2}$, where $SS_{1}=229\times 229$ and $SS_{2}=256\times 256$Step 4: Apply SIFT and ORB on $SS_{1}$Step 5: Apply SIFT and ORB on $SS_{2}$Step 6: Generate texture features from the combination of SIFT and ORBStep 7: Get texture featuresStep 8: Finish

### 3.3. Deep and Prominent Feature Selection Using CNN

A CNN network is designed to mine a large number of features and extract deep and prominent characteristics that can lessen the load and processing power on the classification model. To achieve this, the pretrained dictionary and visually based texture features are combined and fed into the CNN. Several studies [32,33] have used CNN to categorize malware. The CNN model performs better with a variety of information, including text, images, and video files. We use a one-dimensional CNN network containing convolutional layers, pooling layers, dropout layers, and a fully connected layer. Convolution acts as a filter, repeatedly cycling through the combined features and obtaining the best feature representations. Each filter generates a new set of features, called a feature map. The optimal number of filters is determined by adjusting the hyperparameters. We used three convolution layers with 32, 64, and 128 filters, respectively. Max pooling reduces the size of the feature space, the range of features, and the computational cost. This layer also generates a feature map with the most important features from the preceding set. Furthermore, we combine the Keras batch normalization layer with the CNN network. Batch normalization keeps the resultant mean close to zero and the standard deviation close to one. Notably, it operates differently throughout training and testing. This stabilizes the learning process and reduces the number of training epochs deep networks need. In the proposed CNN network, softmax and dropout layers address overfitting. Equation (1) represents the CNN network’s output.

(1) $$o_{k}^{1}=f(c_{k}^{1}+\overset{N_{l-1}}{\underset{i=1}{\sum }}\mathrm{Con}1D(X_{\mathrm{ik}}^{l-1},{\;t}_{i}^{l-1}))$$

where $c_{k}^{1}$ is the parameter bias of the kth neuron in the first layer, $t_{i}^{l-1}$ is the outcome of the ith neuron in layer l − 1, $X_{\mathrm{ik}}^{l-1}$ is the kernel strength from the ith neuron in layer l − 1 to the kth neuron in layer l, and ‘‘f ()” is the activation function. After analyzing the deep features, we chose the top 250 prominent features for accurate malware classification.

### 3.4. Ensemble Model for Malware Classification

The deep and prominent features are fed into the voting-based ensemble model for malware classification and detection.

#### 3.4.1. Naive Bayes (SVM)

To perform classification tasks, the NB algorithm, commonly known as the probabilistic algorithm, is utilized. It is a simple algorithm that works well in a variety of circumstances. The Bayes theorem is utilized to construct the classifier in Equation (2).

(2) $$P\left(y|X\right)=\frac{P\left(X|y\right)P\left(y\right)}{P\left(X\right)}$$

where *y* indicates the class variable, whereas *X* indicates the characteristics or attributes. Here, *X* is defined as (*x*_1_, *x*_2_, …, *x*_n_). Gaussian naive Bayes (GNB) conditional probability arises from normal distribution, as shown in Equation (3).

(3) $$P\left(x_{1}|y\right)=\frac{1}{\sigma_{y}\sqrt{2\pi }}e^{-{\left(x_{i}-\mu_{y}\right)}^{2}}/2\sigma_{y}{}^{2}$$

#### 3.4.2. Support Vector Machine (SVM)

SVM is a supervised learning approach for classification and regression. It classifies by finding the most distinct hyperplane. It locates the hyperplane by widening the distance. Using the kernel function, the kernel trick converts a non-separable job into a separable solution. It is especially useful when dealing with non-linear discrete problems. We used sigmoid as a kernel function. The soft margin of an SVM classifier is calculated by reducing an expression of the kind given in Equation (4).

(4) $$\left[\frac{1}{n}\overset{n}{\underset{i=1}{\sum }}\max\left(0.1-y_{i}\left(w^{T}x_{i}-b\right)\right)\right]+\lambda \|w{\|}^{2}$$

#### 3.4.3. Decision Tree (DT)

Each leaf node in a decision tree represents the outcome, a branch represents a decision rule, and an internal node represents a task. The top node is the root node. It usually segments based on the level of an attribute. A tree is partitioned using iterative segmentation. This flow design could help make better decisions. It uses loss functions to assess the integrity of produced nodes. We employed entropy to estimate the decision node’s impurity, as illustrated in Equation (5).

(5) $$Entropy=-\overset{K}{\underset{i=1}{\sum }}p_{i}\times log_{2}p_{i}$$

The entropy value varies between 0 and 1. The lower the entropy, the higher the purity of the node. Using entropy as a loss function allows for division only if the new nodes tend to have lower entropy than the parent node.

#### 3.4.4. Logistic Regression (LR)

LR accurately predicts binary outcomes (*y* = 0 or 1). LR is better than linear regression for forecasting classification. Equation (6) shows the logistic function.

(6) $$fx=\frac{1}{1+e^{-x}}$$

#### 3.4.5. Random Forest (RF)

RF is an estimator that uses DT models to improve the detection rate and reduce overfitting. DTs are often trained by “bagging”, which creates a “forest” of trees. The bagging technique claims that integrating many DT models will yield excellent performance. During training, it may handle the growth of numerous DTs and extract information, aggregating the results of each DT [34].

#### 3.4.6. Voting-Based Ensemble Learning

Ensemble is a robust model created by systematically combining base technologies. Unlike individual models, the ensemble model is able to solve classification and regression problems. The proposed investigation employs the soft polling ensemble approach. To begin, we used training data to build basic GNB, SVM, DT, LR, and RF models. The efficiency of the base models is then validated using test data, with each model producing a unique classification. To obtain the final classification performance, ensemble learning employs the estimations of several approaches as supplementary information [35]. The trained and texture features are combined for malware classification, as shown in Algorithm 4.
**Algorithm 4:** Malware Classification**Input**: Trained and texture features**Output**: Malware classificationStep 1: Insert T and I
Step 2: $T^{\prime }=\mathrm{CNN}\left(T\right)$ to apply the CNN technique of trained featuresStep 3: $I^{\prime }=\mathrm{CNN}\left(I\right)$ to apply CNN the technique of texture featuresStep 4: Calculate deep PF as a prominent features as a prominent featuresStep 5: Apply voting-based ensemble learning on deep PF
Step 6: App classification as malware or benignStep 7: Finish

Computational complexity is concerned with categorizing computational issues based on their resource utilization and relating these classes to one another. We analyzed the computational complexity for each algorithm presented in Table 1. The complexity is based on the space required for the proposed approach.

## 4. Results and Discussions

### 4.1. Dataset Preparation

The proposed method is thoroughly examined using two datasets obtained from the Canadian Institute for Cybersecurity (https://www.unb.ca/cic/datasets/index.html (accessed on 6 September 2021)). The first dataset, the Canadian Institute of Cybersecurity Android Adware and General Malware (CICAAGM2017) dataset [36] is gathered semiautomatically by installing Android apps on authorized mobile devices. The dataset is generated using 1900 apps and is separated into three classes: adware, general malware, and benign. The adware contains 250 malicious apps, including Airpush, Dowgin, kemoge, mobidash, and shuanet. The general malware consists of 150 malicious apps, including AVpass, fakeAV, fakeflash, GGtracker, and penetho. A total of 1500 apps are included in the benign set. Table 2 contains a detailed description of the dataset. The second dataset, CICMalDroid 2020 [25,37], collected over 17,341 Android samples from different sources, including the VirusTota l service, the Contagio security blog, AMD, and MalDozer between December 2017 and December 2018. The classification of Android apps as malware is critical for cybersecurity investigators to implement effective classification and detection systems. As a result, this dataset contains adware, banking, riskware, and SMS as malware, as well as benign apps. The number of adware, banking, riskware, SMS, and benign apps is 1253, 2100, 2546, 3904, and 1795, respectively. A detailed description of each app is presented in Table 3.

### 4.2. Result Analysis and Performance Comparison

**Figure 5:**
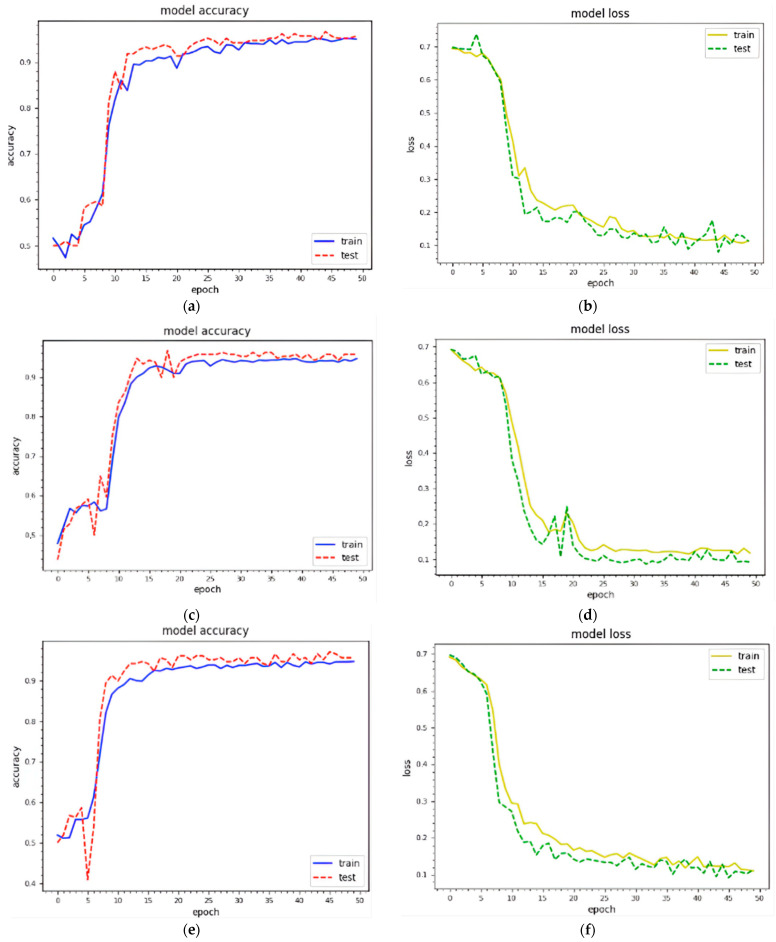

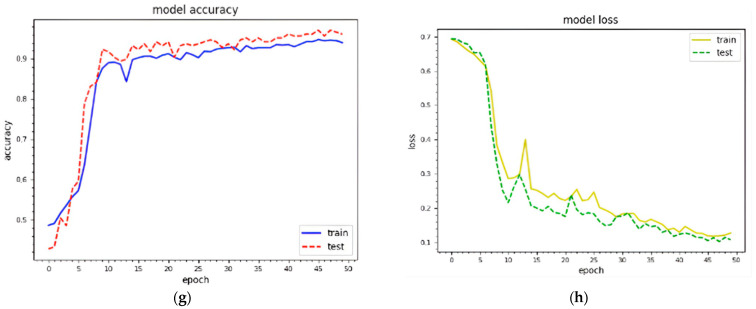
Epoch curves for training and testing data points using different visual representations for **dataset 1**, i.e., 229 × 229, 256 × 256 (training accuracy, training loss; testing accuracy, testing loss). (**a**) 229 × 229 (classification); (**b**) 229 × 229 (classification); (**c**) 229 × 229 (detection); (**d**) 229 × 229 (detection); (**e**) 256 × 256 (classification); (**f**) 256 × 56 (classification); (**g**) 256 × 256 (detection); (**h**) 256 × 256 (detection).

**Figure 6:**
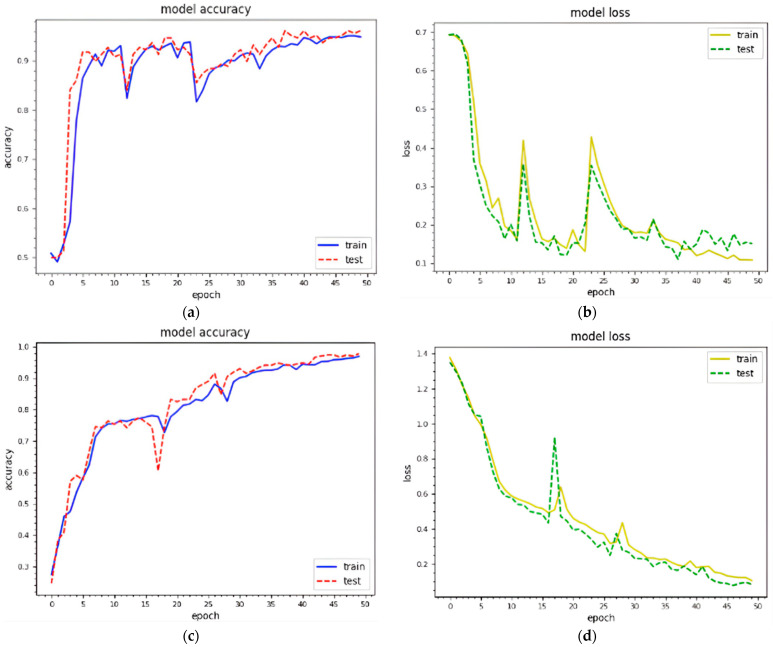

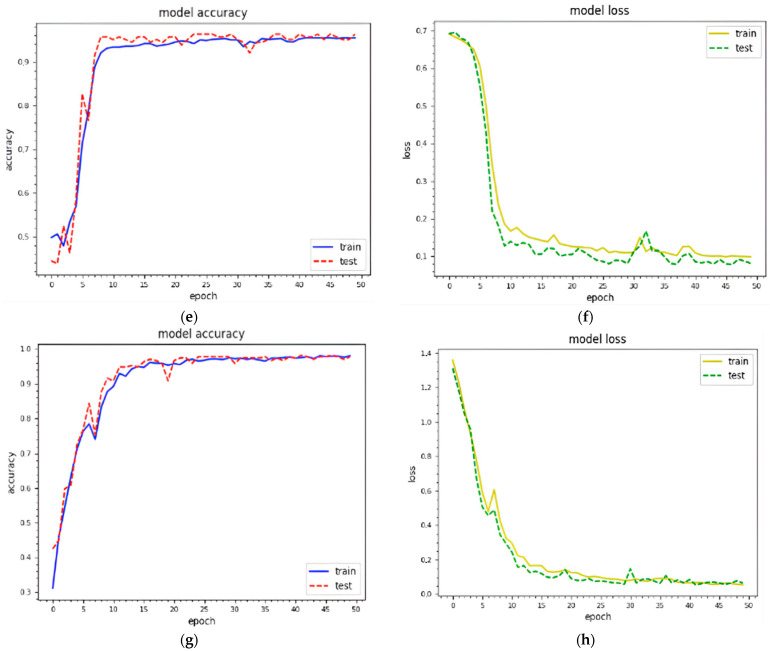
Epoch curves for training and testing data points using different visual representations for **dataset 2**, i.e., 229 × 229, 256 × 256. (training accuracy, training loss; testing accuracy, testing loss). (**a**) 229 × 229 (classification); (**b**) 229 × 229 (classification); (**c**) 229 × 229 (detection); (**d**) 229 × 229 (detection); (**e**) 256 × 256 (classification); (**f**) 256 × 56 (classification); (**g**) 256 × 256 (detection); (**h**) 256 × 256 (detection).

The trained textual features are combined with visual texture features before being fed into the designed model. We generated texture features with 229 × 229 and 256 × 256 and then combined them with textual features to analyze the impact. Figure 5 shows the training and testing curves for malware classification and detection using dataset 1. We utilized two standard image sizes: 229 × 229 and 256 × 256. In terms of model accuracy, the blue and red curves represent the training and testing data points, respectively. In terms of model loss, the yellow and green curves represent the training and testing points, respectively. (a–d) demonstrate classification and detection for 229 × 229 images, whereas (e–h) demonstrate classification and detection for 256 × 256 images. These curves represent the dynamic behavior of the specified model during the training phase. Using 229 × 229 texture features, the model accuracy curves range from 40% to 98% for classification and 40% to 99% for detection. The model accuracy curves for 256 × 256 texture features result in 35% to 98.1% classification and 30%to 99.16% detection accuracy. As a result, the combined features with 256 × 256 texture features outperform. The model loss is inversely proportional to the model accuracy. Figure 6 depicts the training and testing curves for model accuracy and loss using dataset 2. The model accuracy curves achieve between 50% and 98.1% accuracy for classification and between 40% and 99.1% for detection using dataset 1. Similarly, the same curves provide performance accuracy ranging from 30% to 98.11% for classification and from 40% to 99% for detection. It is clear that textual features with 256 × 256 work better for malware detection.

**Figure 7:**
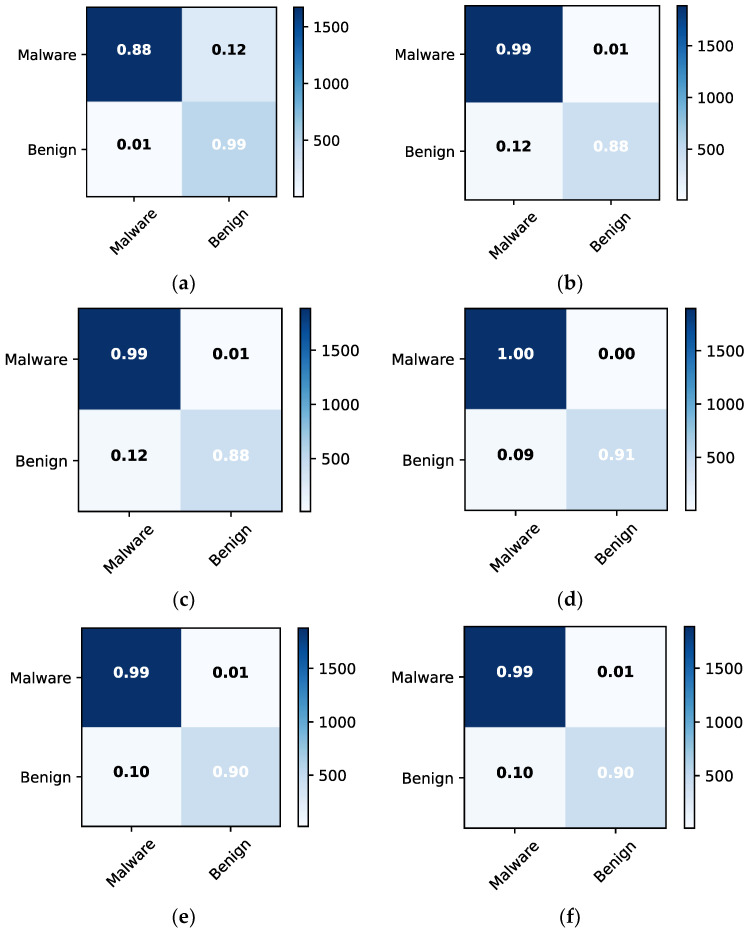
Confusion matrices for malware detection using dataset 2 with 256 × 256. (**a**) GNB; (**b**) SVM; (**c**) DT; (**d**) LR; (**e**) RF; (**f**) ensemble.

**Figure 8:**
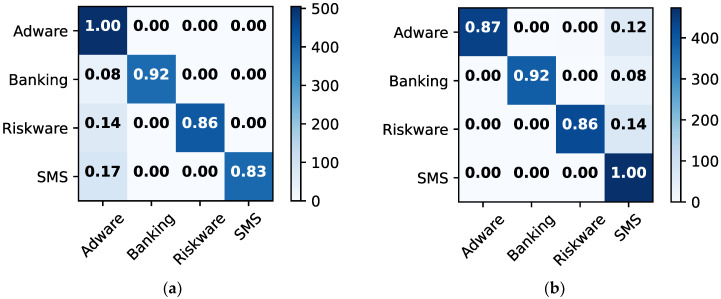

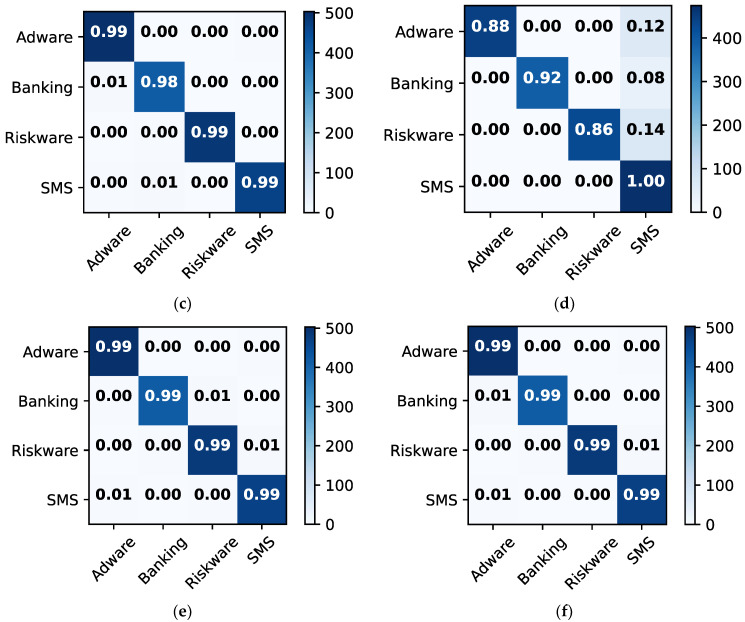
Confusion matrices for malware classification using dataset 2 with 256 × 256. (**a**) GNB; (**b**) SVM; (**c**) DT; (**d**) LR; (**e**) RF; (**f**) ensemble.

The confusion matrices for malware detection are obtained to examine misclassification errors for each class, such as malware and benign. Figure 7 depicts the confusion matrices for the individual approaches and the ensemble model, allowing for detailed comparison. The ensemble model outperforms RF in terms of classification. For instance, both approaches had 99% classification and 12% misclassification accuracy for malware and 90% and 10% for benign, respectively. The LR model behaves similarly to ensemble learning but with different results. For example, LR has a 100% classification accuracy and 0% misclassification for malware and 91% classification and 9% misclassification for benign. Figure 8 depicts the confusion matrices for malware classification using 256 × 256 dataset 2. Ensemble and RF models outperform other methods. For instance, they provide classification and misclassification rates of 99% and 1%, respectively, for each class, such as adware, banking, riskware, and SMS.

Table 4 shows the precision, recall, f1-score, and accuracy measures for both datasets using 229 × 229. Performance matrices are provided for each approach, as well as for the ensemble. The ensemble model outperforms the other models in terms of malware classification and detection when utilizing dataset 1. For malware classification, the precision, recall, f1-score, and accuracy measures are 98%, 97, 98%, and 98.18%, respectively. The same performance measures achieve 99%, 99%, 99%, and 99.02% accuracy for malware and detection, respectively. Using dataset 2, the ensemble approach performs better for malware classification; however, the RF approach works better for malware detection. Malware categorization performance measures are 98, 98%, 98%, and 98.1%, respectively. Similarly, the performance measures for malware detection are 99%, 99%, 99%, and 99.04%, respectively. Table 5 shows the performance measures for malware classification and detection using both 256 × 256 datasets. The proposed approach achieves the best classification results using both datasets with 256 × 256 dimensions. Table 6 shows the malware classification performance measures for each class label using dataset 1. Table 7 shows the performance measures for each class label using dataset 2. The methods with a bold style demonstrate that they outperform others for the designed experiment.

Table 8 depicts the analysis of the optimum features used to determine the best feature selection. The proposed method is tested with a variety of feature counts, such as 100, 150, 200, 250, etc., corresponding to classification accuracy. Dataset 1 is used to examine feature selection with various feature counts. The NB, SVM, DT, LR, RF, and ensemble models provide the highest classification accuracy for 250 features. The classification accuracy increases from 100 to 200 features but decreases after 250. With 400 classification features, classification accuracy increases slightly but then decreases. According to this analysis, 250 is the optimal number of features for the proposed approach.

Generally, classification models produce different results after each execution. To evaluate performance, the datasets are randomly divided into train and test models. As a result, each execution produces unique results for each classification model. We used the same random seed on all classification models with 10 executions to test the scalability and reliability of the proposed ensemble model. Table 9 shows the classification model performance using the same random seeds. On 8 of 10 random seeds, the ensemble model outperforms other classification models, demonstrating that the ensemble model configuration is more reliable than a single classification model. At execution times 2 and 10, the RF slightly outperforms other models relative to the ensemble. Surprisingly, the average performance of 10 executions demonstrates that the ensemble model is more scalable and reliable than the random forest, and it is adopted as the best solution for malware detection and classification. Furthermore, the ensemble model has an accuracy range of 98.98% to 99.02%, whereas the RF has an accuracy range of 98.86% to 99.02%.

Table 10 compares the proposed approach to previously published studies. These studies mostly made use of network traffic to classify Android malware. Aresu et al. [14], showed how analysis of mobile botnets’ HTTP traffic can be utilized to classify them into families. To do so, it analyzes HTTP traffic data to create malware clusters. This method also extracts signatures that can be used to detect new clustered malware with an accuracy of 98.66%. Li et al. [20] presented the Droid Classifier, which automatically builds multiple models over a set of annotated malware apps. Each model is built using common identifiers collected from network traffic. Adaptive threshold settings are designed to represent diverse virus traits with an accuracy of 94.66%. Shanshan et al. [38] proposed identifying infected files by their URLs. Multi-view neural networks provide depth and breadth of information when analyzing malware, in addition to creating and distributing soft attention-weighting elements for use with specific data. The accuracy of URL-based malware classification is 95.74%. Shyong et al. [39] combined static authorization with dynamic network monitoring to classify Android apps. During the dynamic evaluation step, malicious network traces are used to obtain various attributes, and Random Forest is then used to identify malware samples. The average Android malware performance is 98.86%. Shanshan et al. [28] presented a method to detect Android malware using URLs. Multi-view neural networks are used to construct malware detection models that focus on feature depth. The weights of the features are dispersed to work on certain inputs. The suggested approach has an accuracy of 98%. Our technique outperforms this method, with a 99% malware detection accuracy.

The proposed method is thoroughly compared to existing methods using the same datasets. Table 11 shows a performance comparison with state-of-art methods using the same datasets with different strategies. Texture, text, or a combination of both can be used to classify malware. Furthermore, some researchers used a CNN model to classify malware images without using descriptors to select special features. Alani et al. [21] introduced AdStop, a machine-learning-based method that identifies malware in data traffic. The proposed method classified malware using textual features from the CIC-AAGM2017 dataset and a multi-layer perceptron with an accuracy of 98.02%. Acharya et al. [22] proposed a framework *that* extracts clusters using latent Dirichlet allocation and hierarchical clustering techniques. They used a CNN model, which has a precision of 98.3%, to classify malware without relying on any special features. In [22,24,41,42] CNN and TCN models were used to classify malware with texture features. The proposed deep learning models directly collect the malware images for classification without selecting the special features using descriptors. In [21,23,25] multi-layer perceptron (MLP), gradient boosting, and ensemble methods were used to classify malware with textual features. To classify malware, we propose a method that combines textual and texture features from both datasets. When compared to state-of-the-art methods, the proposed approach outperforms, with a classification accuracy of 99%.

### 4.3. Model Interpretation and Validation Using Explainable AI and t-SNE

**Figure 9:**
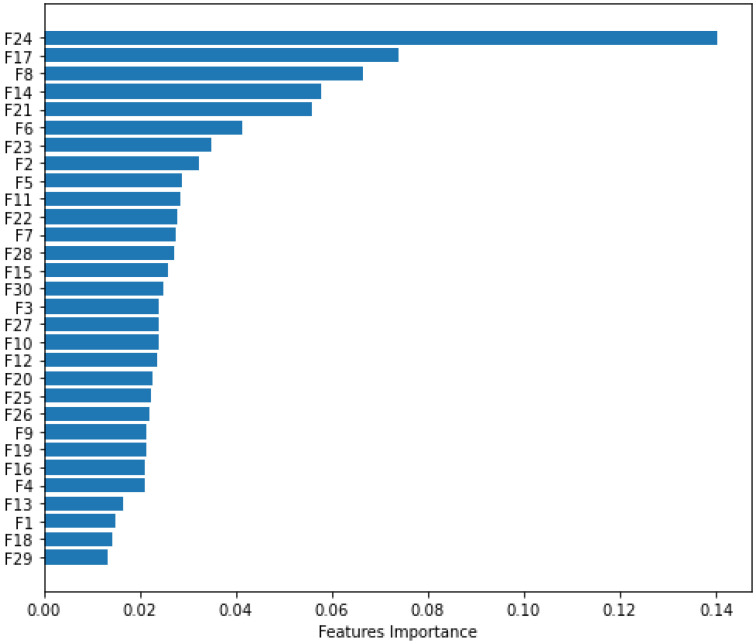
Most significant features.

**Figure 10:**
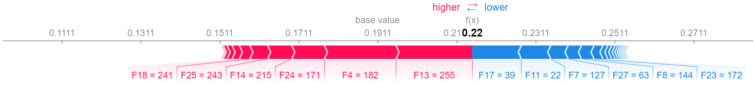
Contribution of features to a certain class based on a threshold value.

**Figure 11:**
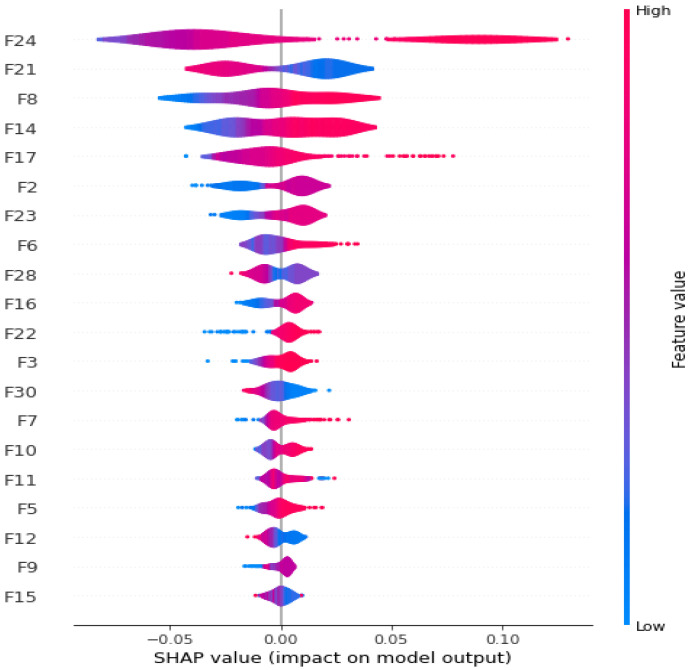
Combined effect of features on model output.

To interpret and validate the proposed approach, we extracted a chunk of the most important features from the embedded matrix. Figure 9 depicts the importance of the features among the 30 features. The feature “F24” is the most effective, indicating that it makes the most contribution to malware classification detection. However, the “F29” feature is the least effective and may perform the worst for the proposed strategy. The “F17” feature is the next most effective feature. Thus, we can readily determine which features are the most and least important. To explain the impact of each feature on the model output, we used the Local Interpretable Model-agnostic Explanation (LIME) and SHapley Additive exPlanations (SHAP) libraries [43]. Figure 10 illustrates the proportionate contribution of features to from the average of samples with a base value of 0 (malware) to the output value of 1 (benign). The values for this sample are indicated by numbers at the bottom of the figure. In our case, the base value is 0.22. The red values are those that are moving underneath the base value, whereas the blue values are those that are moving above the base value. The base value is a threshold, and values less than the base value can contribute to the malware class. Values that are greater than the base value can contribute to the benign class. This allows us to evaluate the contribution of each feature to a specific class. Figure 11 depicts the effect of combined features on model output. The red color represents a higher contribution of each feature, whereas the green color represents smaller contributions. The combined effect of the “F24” feature is significant, whereas that of F15 is the smallest. This allows us to easily describe the impact of each feature on a certain class, such as malware or benign. This experiment evaluates the effectiveness of each feature, providing a clear picture of how each attribute affects the model output.

**Figure 12:**
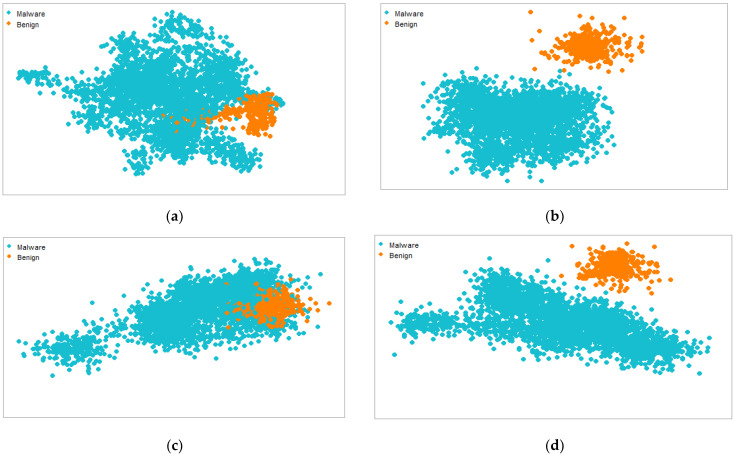
t-SNE visualization for fused features using minimum (30 and 35) and optimal (50 and 70) perplexity values. (**a**) Perplexity, 30; (**b**) perplexity, 50; (**c**) perplexity, 35; (**d**) perplexity, 70.

The purpose of the t-distributed stochastic neighbor embedding (t-SNE) visualization method is to identify whether features possess high or sparse knowledge. Furthermore, the t-SNE method is intended to evaluate the efficiency of the suggested approach. Maaten et al. [44] proposed the t-SNE method to visualize high-dimensional data. Figure 12 shows the attentive ratio of semantic and syntactic feature local and global scores for various perplexity values. Using the R programming language, we designed two t-SNE visual studies. In the first experiment, we attempted to determine how much perplexity is required to distinguish between the benign and malicious classes. The best Android malware clusters are distinguished by the highest perplexity scores in the second experiment. For instance, (a,c) have the lowest perplexity values, whereas (b,d) have the highest values. t-SNE makes use of iterations to distinguish between different types of samples. We utilized 400 iterations for each perplexity factor to display the distinct malware and benign groupings. The dataset density has a significant impact on the overall classification results. Because more qualitative data are presented for training, a higher density usually improves accuracy. To improve classification outcomes, the t-SNE visual clusters are better segregated using optimal perplexity settings. A dataset can be divided into sections using an acceptable perplexity value and classified using important hyperparameters. This method is used to demonstrate the efficacy of the presented strategy because semantic aspects can be extracted and classified as malware or benign to improve classification performance.

## 5. Conclusions

Mobile apps are susceptible to malicious network activity because of their frequent remote access. Such threats could gather crucial information while adversely affecting commerce, social order, and financial institutions. The malware detection system used in this study takes advantage of the combined influence of textual and textural features, combining the strengths of text and visual elements. We proposed an algorithm for a packet parser that is used to collect HTTP and TCP streams from the encrypted communications generated by malicious traffic. It is possible to recover training vocab features from decoded information using word2vec embeddings. A method for transforming malware images is then developed to examine the byte stream with visual features. We used two standard image sizes (229 × 229) and (256 × 256) to test the proposed approach on features of varying size. The texture features from malware images are combined with trained vocab to classify and detect malware. We designed a voting-based ensemble model for accurate malware classification and detection. The classification and detection rates for dataset 1 with an image size of 229 × 229 are 98.18% and 99.02%, respectively. The classification and detection rates for dataset 2 using a 229 × 229 image size are 98.1% and 99.04%, respectively. Similarly, for a 256 × 256 image size with dataset 1, these values are 96% and 99%, respectively. For dataset 2, these values are 98.11% and 99%, respectively. The first dataset with an image size of 229 × 229 provides better classification results than the second dataset with an image size 256 × 256. The proposed approach outperforms the state-of-the-art methods using the same datasets, as shown in Table 9 and Table 11.

In the future, we plan to extract the trained vocab from other pretrained models, such as FastText and BERT. Then, the trained features can be combined with texture features to classify malware. Moreover, the proposed method can be tested with different types of ensembles, such as bagging and stacking.

## Figures and Tables

**Table 1:** Computational cost analysis.

Algorithm	Computational Costs		
	P/T	D/E/D	C/S
**Algorithm 1**	$|P|=\left|p_{1}\left|+\right|p_{2}\right|+\ldots$ $+|p_{n}|$	|P| = |n|	$\left|P^{\prime }\right|$ = |n|
**Algorithm 2**	|HTTP| + |TCP|	|TF|	|MTF|
**Algorithm 3**	$|B|=\left|B_{1}\left|+\right|B_{2}\right|+\ldots$ $+|B_{n}|$	$\left|I|+|SS_{1}\left|+\right|SS_{2}\right|$	$\left|SS_{1}\left|+\right|SS_{2}\right|$
**Algorithm 4**	|T| + |I| = |n|	$|T^{\prime }|+$$\left|I^{\prime }\right|$= |n|	|PF|

P/T, packets/tokenize; D/E/D, decryption/extraction/decomposed, TF, trained feature; MTF, mining trained files; C/S, computes/shifting.

**Table 2:** Android Adware and General Malware Dataset (CIC-AAGM2017) (dataset 1).

App	No. of Apps	Family	Description
Adware	250	Airpush	Distributes intrusive adverts to bypass security
		Dowgin	Ad package that collects data
		Kemoge	Takes over the user’s Android phone
		Mobidash	Created to broadcast ads and illegal access
		Shuanet	Takes over the user’s device
General Malware	150	AVpass	A utility software masquerading as a clock
		FakeAV	Phishing scam to obtain full-version apps
		FakeFlash	Fake Flash software that redirects viewers to a fake website
		Ggtracker	Employed to obtain data via SMS fraud
		Penetho	Fake tool to recover WiFi passwords
Benign	1500	Benign	Clean apps (not malicious)

**Table 3:** CICMalDroid 2020 dataset (dataset 2).

App	Family	No. of Apps	Description
Malware	Adware	1253	Ads can be hidden within malware-infected programs
	Banking	2100	Connects directly to the user’s online payments
	Riskware	2546	Any legitimate program can be abused to inflict harm
	SMS	3904	Attacks via SMS
Benign	Benign	1795	Clean apps (not malicious)

**Table 4:** Performance comparisons for malware classification and detection using both datasets with 229 × 229.

Dataset 1 (229 × 229)					
	Methods	Precision (%)	Recall (%)	F1-Score (%)	Accuracy (%)
Classification	GNB	86	90	86	87.21
	SVM	95	89	81	93.11
	DT	98	98	97	98.03
	LR	95	89	91	93.34
	RF	98	97	98	98.03
	**Ensemble**	**98**	**97**	**98**	**98.18**
Detection	GNB	94	91	92	92.24
	SVM	93	93	92	92.16
	DT	99	99	98	98.94
	LR	93	93	92	92.16
	RF	99	99	99	99.02
	**Ensemble**	**99**	**99**	**99**	**99.02**
**Dataset 2 (229 × 229)**					
Classification	GNB	98	95	96	98.02
	SVM	96	92	94	95.96
	DT	95	95	94	95.04
	LR	98	97	97	97.98
	RF	97	97	97	97.02
	**Ensemble**	**98**	**98**	**98**	**98.1**
Detection	GNB	94	93	93	93.11
	SVM	93	91	91	91.08
	DT	99	99	99	98.96
	LR	95	93	93	93.1
	**RF**	**99**	**99**	**99**	**99.04**
	Ensemble	95	93	93	94.16

**Table 5:** Performance comparisons for malware classification and detection using both datasets with 256 × 256.

Dataset 1 (256 × 256)					
	Methods	Precision (%)	Recall (%)	F1-Score (%)	Accuracy (%)
Classification	GNB	91	84	85	84.98
	SVM	92	91	90	91.02
	DT	96	96	96	96
	LR	92	91	90	91.02
	RF	96	96	96	96
	**Ensemble**	**96**	**96**	**96**	**96**
Detection	GNB	94	94	94	94.01
	SVM	94	94	94	94.01
	DT	98	98	97	98
	LR	94	95	94	94.08
	**RF**	**99**	**99**	**99**	**99**
	Ensemble	94	95	94	94.11
**Dataset 2 (256 × 256)**					
Classification	GNB	93	90	91	91.14
	SVM	97	98	97	97.21
	DT	96	96	96	96.1
	LR	98	98	97	97.99
	RF	97	97	97	97
	**Ensemble**	**98**	**98**	**99**	**98.11**
Detection	GNB	93	90	91	90.84
	SVM	93	91	91	91.46
	DT	99	99	99	99
	LR	94	91	92	91.36
	RF	99	99	99	99
	**Ensemble**	**99**	**99**	**99**	**99**

**Table 6:** Per-class performance comparisons for malware classification using dataset 1 with 256 × 256.

Class	Method	Precision (%)	Recall (%)	F1-Score (%)
Adware	GNB	88	100	94
	SVM	88	100	94
	DT	97	98	98
	LR	88	100	94
	**RF**	**97**	**100**	**99**
	Ensemble	88	100	94
Gen: Mal	GNB	100	88	93
	SVM	100	88	93
	DT	98	97	98
	LR	100	88	93
	**RF**	**100**	**98**	**99**
	Ensemble	100	88	93

**Table 7:** Per-class performance comparisons for malware classification using dataset 2 with 256 × 256.

Class	Method	Precision (%)	Recall (%)	F1-Score (%)
Adware	GNB	73	100	85
	SVM	100	87	93
	DT	98	99	99
	LR	1	88	93
	**RF**	**99**	**99**	**99**
	Ensemble	98	99	99
Banking	GNB	100	92	96
	SVM	100	92	96
	DT	99	98	98
	LR	1	92	96
	RF	99	99	99
	**Ensemble**	**100**	**99**	**99**
Riskware	GNB	100	86	93
	SVM	99	86	92
	DT	99	99	99
	LR	1	86	93
	RF	99	99	99
	**Ensemble**	**100**	**99**	**99**
SMS	GNB	100	83	91
	SVM	74	100	85
	DT	99	99	99
	LR	74	1	85
	**RF**	**99**	**99**	**99**
	Ensemble	98	99	99

**Table 8:** Optimum feature analysis.

Dataset 1 (229 × 229)									
Method	Features								
	100	150	200	250	300	350	400	450	500
NB	90.61	91.53	92.14	**92.24**	91.22	90.52	90.66	89.71	89.62
SVM	91.18	91.74	91.36	**92.16**	91.49	91.92	91.82	90.28	89.12
DT	95.84	96.51	97.36	**98.94**	97.44	96.62	97.88	95.72	95.14
LR	91.56	91.92	91.98	**92.16**	92.14	91.94	92.1	90.82	90.24
RF	97.24	97.54	98.82	**99.02**	98.13	97.76	96.71	96.19	95.96
Ensemble	96.88	97.76	98.96	**99.02**	98.52	97.48	96.98	96.71	96.28

**Table 9:** Average performance comparison with multiple executions.

Dataset 1 (229 × 229)											
Method	Execution Times										
	1	2	3	4	5	6	7	8	9	10	Average
NB	92.14	92.24	92.2	91.99	92.22	92.18	92.24	92.22	92.24	91.98	**92.16**
SVM	92.12	92.16	92.16	91.99	92.11	91.98	91.92	92.16	92.12	92.14	**92.09**
DT	98.88	98.94	98.9	98.94	98.44	98.92	98.89	98.72	98.94	98.92	**98.85**
LR	92.16	92.16	92.14	92.04	92.1	91.99	91.96	92.16	92.13	92.16	**92.1**
RF	98.97	**99.02**	98.96	99.02	99.01	98.74	98.91	98.86	99.00	**99.02**	**98.95**
Ensemble	**98.99**	99.00	**98.99**	99.02	**99.02**	**99.00**	**99.00**	**98.98**	**99.02**	99.00	**99.01**

**Table 10:** Comparison with previously published works.

Work	Year	Method	Dataset	Accuracy (%)
**Aresu et al. [14]**	2015	Signature-based clustering	Drebin and VirusTotal	96.66
**Li et al. [40]**	2016	Droid classifier	VirusTotal	94.66
**Shanshan et al. [38]**	2018	Skip gram with neural network	Malicious URLs (Emulator)	95.74
**Shanshan et al. [13]**	2019	C4.5 decision tree	Drebin and VirusTotal	97.89
**Shyong et al. [39]**	2020	Random forest	Drebin	98.86
**Shanshan et al. [28]**	2020	Multi-view neural network	VirusShare	98.81
**Our approach**	…	Hybrid features with ensemble learning	CIC-AAGM2017 and CICMalDroid 2020	99

**Table 11:** Performance comparison with state-of-the-art methods using the same datasets.

Work	Dataset	Strategy	Method	Accuracy (%)
**Alani et al. [21]**	CIC-AAGM2017	Textual	MLP (DNN)	98.02
**Acharya et al. [22]**	CIC-AAGM2017	Texture	CNN	98.3
**Hadiprakoso et al. [23]**	CICMalDroid 2020	Textual	Gradient Boosting	96.35
**Mohammad et al. [41]**	CICMalDroid 2020	Texture	CNN	96.4
**Zhang et al. [24]**	CICMalDroid 2020	Texture	TCN	95.44
**Mahdavifar et al. [25]**	CICMalDroid 2020	Textual	Ensemble	97.84
**Peng et al. [42]**	CICMalDroid 2020	Texture	CNN	98.6
**Our approach**	CIC-AAGM2017 & CICMalDroid 2020	Hybrid	Hybrid features with ensemble learning	99

## Data Availability

The data that support the findings of this study are openly available in the Canadian Institute for Cybersecurity—Android Adware and General Malware Dataset (CIC-AAGM2017) and CICMalDroid 2020 at https://www.unb.ca/cic/datasets/android-adware.html (accessed on 6 September 2021) and https://www.unb.ca/cic/datasets/maldroid-2020.html (accessed on 6 September 2021), respectively.
